# Standardized Definition of Red Flags in Musculoskeletal Care: A Comprehensive Review of Clinical Practice Guidelines

**DOI:** 10.3390/medicina61061002

**Published:** 2025-05-28

**Authors:** Lorenzo Storari, Jennifer Piai, Mirko Zitti, Graziano Raffaele, Fabio Fiorentino, Rachele Paciotti, Fabiola Garzonio, Giulia Ganassin, James Dunning, Giacomo Rossettini, Daniel Feller, John D. Heick, Firas Mourad, Filippo Maselli

**Affiliations:** 1 Department of Human Neurosciences, Sapienza University of Rome, 00185 Rome, Italy; ftstorari@gmail.com (L.S.); jennifer.piai.jp@gmail.com (J.P.); mirkozitti89@gmail.com (M.Z.); graziano.raffaele997@gmail.com (G.R.); fiorentinof02@gmail.com (F.F.); rachele.paciotti@gmail.com (R.P.); giuliagana@yahoo.it (G.G.); giacomo.rossettini@gmail.com (G.R.); 2 American Academy of Manipulative Therapy Fellowship in Orthopaedic Manual Physical Therapy, Montgomery, AL 36104, USA; james.dunning@spinalmanipulation.org; 3 Montgomery Osteopractic Physical Therapy & Acupuncture Clinic, Montgomery, AL 36104, USA; 4 School of Physiotherapy, University of Verona, 37129 Verona, Italy; 5 Department of Physiotherapy, Faculty of Spor Sciences, Universidad Europea de Madrid, Calle Tajo s/n, 28670 Villaviciosa de Odon, Spain; 6 Provincial Agency for Health of the Autonomous Province of Trento, 38100 Trento, Italy; danielfeller.ft@gmail.com; 7 Department of General Practice, Erasmus MC, University Medical Centre, 3015 CA Rotterdam, The Netherlands; 8 Department of Physical Therapy, Northern Arizona University, Flagstaff, AZ 86011, USA; john.heick@nau.edu; 9 Department of Health, LUNEX University of Applied Sciences, L-4671 Differdange, Luxembourg; firas.mourad@me.com; 10 Luxembourg Health & Sport Sciences Research Institute A.s.b.l, L-4671 Differdange, Luxembourg; 11 Sovrintendenza Sanitaria Regionale Puglia INAIL, 70126 Bari, Italy

**Keywords:** musculoskeletal diseases, clinical practice guidelines, diagnosis, differential, referral, signs and symptoms

## Abstract

*Background and Objectives:* The aging population and the COVID-19 pandemic have led to a rise in severe conditions, including musculoskeletal (MSK) disorders. Although MSK conditions are often managed in primary care, they may sometimes mask serious illnesses requiring urgent diagnosis. The red flag (RF) concept is essential for identifying signs and symptoms of potentially severe disease. However, RF criteria vary across clinical guidelines and lack consistency. With the growing role of direct access to physiotherapy—bypassing physician referral—physiotherapists must develop strong differential diagnostic skills to identify serious pathologies that mimic MSK disorders. This review aims to systematically map how RFs are defined in MSK clinical practice guidelines (CPGs), supporting the move toward a standardized definition for clinical and research use. *Materials and Methods:* A comprehensive literature search was conducted in PubMed, Web of Science, Scopus, and Cochrane databases. Included studies were CPGs and systematic reviews (SRs) of CPGs addressing MSK disorders and incorporating the RF concept. Data extraction followed a rigorous process, and RF definitions were synthesized and compared in table format. *Results:* Out of thirteen-thousand three-hundred and ninety-three articles identified, fourteen met inclusion criteria (seven CPGs and seven SRs of CPGs), spanning both physiotherapy and medical fields. All definitions described RFs as signs or symptoms indicating possible serious pathology requiring further investigation or referral. Some definitions referred broadly to “patterns of signs or symptoms”, while others offered more precise criteria. *Conclusions:* This review highlights the lack of a standardized RF definition in MSK care, leading to inconsistencies in clinical decision-making and diagnosis. To improve patient safety and guide clinicians—especially in direct-access contexts—a unified, internationally recognized definition of RFs is needed in future guidelines.

## 1. Background

The continued aging of the global population is expected to further increase the prevalence of severe pathologies, a trend that has been exacerbated by the COVID-19 pandemic, as reported by the 2021 Global Burden of Disease Study [1,2]. Severe pathologies have been defined as life-threatening or severely disabling conditions, or those requiring immediate (within 48 h) or urgent (within 30 days) medical attention [3]. Unfortunately, such diseases may sometimes masquerade as musculoskeletal (MSK) disorders [1,4]. MSK disorders are typically managed in primary care settings [5] and are often treated conservatively; however, certain presentations may require surgical intervention or reflect systemic diseases that demand specific treatments or thorough diagnostic evaluation [6]. Despite the growing adoption of direct access physiotherapy clinics in high-performing healthcare systems due to its safety, cost-effectiveness, and significant economic benefits in patient management [5,7], primary healthcare professionals often underestimate the risk of encountering severe pathology in musculoskeletal (MSK) patients, largely because such conditions are relatively rare [1]. Nevertheless, the likelihood of complex cases with multiple comorbidities is increasing, which in turn raises the potential for encountering serious pathologies [2]. In response to this evolving clinical landscape, a growing number of healthcare professionals are now actively screening for RFs [1,8], namely clinical indicators of severe underlying conditions that should prompt further evaluation or diagnostic testing before initiating treatment [9]. The diagnostic utility of many RFs is unknown [8,10]. Combining multiple RFs has been shown to improve diagnostic accuracy [9]. Although recognizing red flags (RFs) is essential for avoiding inappropriate interventions and ensuring timely and appropriate clinical decisions, there remains a significant lack of consensus regarding their definition and application across clinical guidelines. This inconsistency can contribute to unnecessary and costly diagnostic procedures, as well as suboptimal clinical decision-making [11,12]. The absence of a clear and universally accepted definition of RFs in the literature further complicates their use in practice, particularly for healthcare professionals operating in direct access settings [13]. The current heterogenous definition for RFs hinders the accurate application of screening for referral to other healthcare professionals (physicians or hospitals for urgent cases) and the development of clinical practice guidelines [8]. Although severe pathologies underlying MSK conditions are infrequent, physiotherapists who are in first-contact roles are increasing [13]; therefore, for patient safety, it is crucial that these encounters are immediately recognized and referred in a timely fashion for medical or surgical attention. Direct access to physiotherapy streamlines patient pathways, reducing the burden on general practitioners and potentially avoiding unnecessary referrals [13]. Consequently, enhanced theoretical and practical knowledge is essential for improving clinical practice and ensuring favorable outcomes for patients with serious pathologies [1]. This comprehensive review aims to systematically map and summarize the literature on the varying RF definitions within MSK clinical practice guidelines. A unified operational definition for RFs would likely assist researchers and clinicians in establishing their clinical utility.

## 2. Methods

### 2.1. Protocol and Registration

This comprehensive review was based on the model suggested by the updated methodological guidelines of the Joanna Briggs Institute (JBI), recognized for its rigorous standards in evidence synthesis. To ensure transparent and detailed reporting, we followed the indications outlined in the 2024 JBI Manual for Evidence Synthesis [14], despite the absence of established checklists for comprehensive review reporting. A protocol was prospectively registered in the OSF Registries of the Center for Open Science (https://accounts.osf.io, accessed on 3 December 2024) under registration number 10.17605/OSF.IO/FK532.

### 2.2. Inclusion Criteria

Studies were eligible for inclusion based on the Population, Concept, and Context (PCC) framework:

- Population: individuals of any age with MSK disorders.
- Concept: systematic reviews of clinical practice guidelines and clinical practice guidelines that explicitly reported a definition of RFs.
- Context: musculoskeletal healthcare settings.

Only articles published in English, Spanish, and Italian were considered.

### 2.3. Exclusion Criteria

Studies that did not meet the aforementioned inclusion criteria or did not provide a clear definition of RFs were excluded from this review.

### 2.4. Search Strategy

A comprehensive literature search was conducted across the following databases up to 1 April 2024: PubMed, Web of Science, and Scopus. Additionally, Google Scholar was utilized to identify further relevant documents and the grey literature. Search strategies were customized for each database, incorporating MeSH terms (where applicable) combined with Boolean operators (AND, OR, NOT). No restrictions were applied regarding publication date. The complete search strategy for each database is detailed in Appendix A.

### 2.5. Study Selection

Duplicate records were automatically removed using Rayyan software (Ver. 1.6.1, 2024, Qatar Computing Research Institute, Qatar). The study selection process was performed independently by two reviewers (JP and LS) under the supervision of a third reviewer (FMa), involving dual analysis using the Rayyan QCRI web application [15]. Both reviewers possessed expertise in MSK disorders. Retrieved documents were screened in Rayyan through a two-stage process: title and abstract review, followed by full-text reading. Reasons for exclusion were documented. Full-text articles meeting the initial criteria were further examined to identify all reported definitions of the term “RF” within the context of differential diagnosis or physical examination for MSK disorders. A comprehensive list of the studies that were excluded during title and abstract screening is available in the Appendix A.

### 2.6. Data Extraction

Two reviewers (RP and LS) performed data extraction independently using a pre-defined, standardized data extraction form. Any discrepancies were resolved through discussion, with the involvement of a third reviewer (FMa) if necessary. Extracted information encompassed general study characteristics (author, publication year, study design, country, setting), population details, and specific definitions of the term “RF” in the context of MSK disorders. The following data points were recorded for each included study:

1. Author and year of publication;
2. Study design;
3. Definitions and application of RFs;
4. Any other pertinent information for the analysis.

Disagreements between the two primary reviewers were resolved through consensus-based discussion, and the third reviewer was consulted when consensus could not be reached. All extracted data were compiled into an electronic database to facilitate subsequent analysis.

### 2.7. Agreement

Cohen’s kappa (K) was used to assess the interrater agreement between the two authors (FM, AC) for full-text selection (K = 0.78; 0.61–0.80 IC 95%). Cohens’ K was interpreted according to Altman’s definition: k < 0.20 poor, 0.20 < k < 0.40 fair, 0.41 < k < 0.60 moderate, 0.61 < k < 0.80 good, and 0.81 < k < 1.00 excellent [16].

### 2.8. Data Synthesis

Data were reported qualitatively. To synthesize the extracted data and identify the precise definitions of RFs, a summary table was created to highlight the similarities and differences across the various definitions reported in the included studies.

## 3. Results

**Figure 1:**
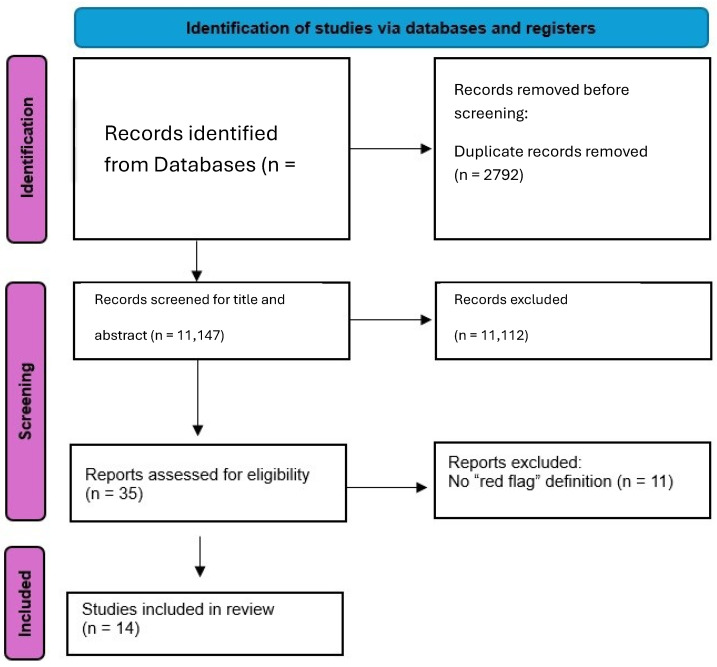
Flow diagram.

A total of fourteen full text articles were included (seven clinical practice guidelines and seven systematic reviews of clinical practice guidelines). The selection process is described in Figure 1. Cohen’s kappa (k) for inter-reviewer agreement ranged from 0.8 to 1.0, indicating a high level of agreement. The analysis of the seven included CPGs revealed a consistent conceptualization of RFs as clinical indicators prompting the consideration of a serious underlying pathology and the potential need for further medical or surgical action (Table 1). Specifically, Ladeira (2011) [17] defined RFs in the context of low back pain as identifiers of patients requiring specialist referral for conditions such as cancer, infection, cauda equina syndrome, fracture, and vascular issues, emphasizing the importance of considering RF clusters. Similarly, Cote et al. (2016) [18] outlined RFs for neck pain as risk factors for serious pathologies like cancer, infection, and fractures, warranting further investigation and referral, utilizing the Canadian C-spine Rule for trauma cases. In the domain of primary care for headaches, Dowson et al. (2002) [19] employed the terms “sinister headache” and “headache alarms” as RFs necessitating specialist referral, focusing on changes in headache patterns and associated alarming features. For nonarthritic hip joint pain, Enseki et al. (2014) [20] defined RFs as clinical indicators suggesting more serious or unrelated conditions, particularly when the presentation deviates from typical patterns or lacks improvement with standard interventions. Within the context of specific musculoskeletal conditions, Peter et al. (2011) [21] and Hurkmans et al. (2011) [22] defined RFs in hip/knee osteoarthritis and rheumatoid arthritis, respectively, as signs and symptoms indicating potentially serious underlying conditions requiring prompt attention and possible referral. Finally, in their guideline for low back and radicular pain, Van Wambeke et al. (2020) [23] underscored the critical role of evaluating patients to exclude RFs, defined as signs and symptoms of serious underlying pathology, and highlighted the clinical significance of RF clusters in this process. Regarding the analysis of the seven systematic reviews of clinical practice guidelines, the present comprehensive review revealed a focus on identifying, comparing, and evaluating the application of RFs across existing guidelines for musculoskeletal conditions. Verhagen et al. (2016) [24] conducted a broad review of low back pain guidelines, identifying a substantial number of distinct RFs and categorizing them according to underlying serious pathologies such as malignancy, fracture, infection, and cauda equina syndrome, also noting RFs not specific to these categories. In a more focused review, Verhagen et al. (2017) [25] specifically examined malignancy-related RFs in low back pain guidelines, defining them as signs or symptoms signaling serious pathology and listing both endorsed and non-endorsed RFs for this condition. Similarly, O’Connell et al. (2016) [26] reviewed low back pain guidelines, observing a general recommendation to consider alternative diagnoses, but highlighted a lack of detailed and consistent methods for RF screening across the reviewed guidelines. Notably, the Canadian guideline was noted as an exception for its specific MRI indications based on certain RF presentations. While presenting a clinical practice guideline for neck pain, Bier et al. (2018) [27] also reviewed existing evidence on RFs for this region, defining them as warning signs of serious pathology requiring referral. They pointed out the weak and inconsistent evidence base for many neck pain RFs due to their generic nature and high false positivity rates, while listing potential serious pathologies. Parreira et al. (2019) [28] specifically reviewed guidelines concerning RFs for fracture in low back pain, defining RFs as clinical indicators raising suspicion of serious pathology. They identified commonly endorsed RFs for fracture but emphasized the greater diagnostic utility of RF combinations over individual indicators. Likewise, in their guideline for low back pain, Bussières et al. (2018) [29] presented a list of RFs indicative of serious structural or systemic pathologies. Finally, Feller et al. (2024) [12] conducted a comprehensive systematic review of neck pain guidelines, identifying many RFs which they categorized by potential serious pathologies, underscoring the breadth of RFs considered across different guidelines for neck pain. For a detailed description of RF definitions across full texts, see Table 1.

## 4. Discussion

This review aimed to address the lack of a universally accepted definition for RFs in the MSK context, emphasizing the potential negative impact of this ambiguity in clinical practice, leading to delayed diagnoses, misdiagnoses, or inadequate treatment. The findings underscore the variability in how RFs are defined and applied, with some definitions being broad and others more specific. While most of the literature agrees on the role of RFs as signs or symptoms indicative of serious, life-threatening conditions, there remains a lack of consensus on the specific symptoms that should be categorized as RFs and the subsequent steps to take after their identification. Despite widespread use in clinical practice, the inconsistency in RF definitions contributes to challenges in interpretation and application, potentially compromising patient care. As highlighted by the Standards of Physiotherapy Practice (2011) [30], physiotherapists are required to perform independent assessments to determine whether a patient is suitable for physiotherapy treatment. These assessments should be based on a thorough evaluation of the patient, including risk assessments and close collaboration with other healthcare professionals. However, the existing guidelines do not consistently provide clear and standardized definitions for RFs, resulting in variability in practice. Conversely, other study designs—such as conceptual frameworks or position statements, which do not occupy the top of the evidence hierarchy [31], appear to align more closely with the current definition of red flags and reflect best practices for screening and referral [8,32]. However, this presents a challenge for clinicians, who are expected to stay updated using the most accessible and efficient tools available, namely clinical practice guidelines [31]. The evidence also suggests that the RF screening tools may lack sufficient sensitivity and specificity to accurately diagnose and appropriately rule out or rule in the suspected sinister conditions. When combined with low condition prevalence, the post-screening probability remains minimal, which represents a significant limitation in both clinical guidelines and daily practice [26]. This highlights the need for improved screening tools that can more effectively identify the presence of serious conditions. One of the main goals of this review was to identify and compare various RF definitions in musculoskeletal contexts, with a focus on clinical practice guidelines (CPGs). Although the definitions of RFs generally describe them as indicators of serious conditions, they differ in terms of specificity. Some guidelines broadly describe RFs, while others list specific signs or symptoms. These differences underscore the importance of establishing a unified, standardized definition to improve clinical practice consistency.

Across both CPGs and systematic reviews of guidelines, a consistent operational definition of RFs emerges, according to which RFs are clinical features that alert the clinician to the potential presence of a serious underlying pathology beyond typical musculoskeletal conditions. RFs may include subjective comments from the patient, specific signs that are observed by the clinician, or tests and measures that help to identify the serious condition. The primary purpose of identifying RFs is to guide decisions regarding the need for further medical investigation, imaging, pharmacological treatment, urgent surgical intervention, or referral to other healthcare professionals. Our findings align with the systematic review by Henschke et al. (2013) [33], which assessed the diagnostic performance of clinical features (“red flags”) for spinal malignancy in low back pain. Their review identified a comprehensive list of potential RFs, including age > 50 and >70, constant progressive pain, previous cancer history, unexplained weight loss, and systemic symptoms, many of which were also listed in the CPGs analyzed in our review [17,18]. However, Henschke et al. (2013) [33] focused on the diagnostic accuracy of these individual RFs, a point echoed by O’Connell et al. (2016) [26] in their critique of the limited sensitivity and specificity of RFs. Similarly, the systematic review by Han et al. (2023) [34] on red flags for vertebral fracture in low back pain identified several key RFs, such as older age (>50, >70, or >74), trauma, corticosteroid use, and neurological signs. These findings are consistent with the RFs listed in the CPGs included in the current review [17,18,29]. Additionally, Han et al. (2023) [34] highlighted the potential role of RF combinations, a concept also emphasized in some of the CPGs we analyzed [17,23,28]. This emphasis on RF clustering is further supported by the systematic review by Maselli et al. (2022) [9] on thoracolumbar pain, which also investigated the diagnostic value of RFs and likely underscored the importance of considering RFs in combination rather than in isolation to improve diagnostic accuracy.

Based on the common elements found in the definitions across the included studies, including the CPGs and the three additional systematic reviews, we propose the following unified definition of RFs: “Red flags are specific signs or symptoms that, when present during the patient’s history or physical examination, raise the level of suspicion of an underlying serious or life-threatening condition that may warrant referral for (immediate or urgent) medical attention. These signs and symptoms include, but are not limited to, risk factors for cancer, vertebral fractures, severe infections, and other systemic pathologies”.

This definition aims to standardize the concept of RFs in musculoskeletal clinical practice, providing clear guidance for physiotherapists and other healthcare providers in the early identification of potentially life-threatening conditions, which could be used as a preliminary step to conduct further studies (e.g., a Delphi consensus). However, this proposed definition also highlights a significant limitation in current RF usage, that is, while RFs are useful screening tools, they do not offer clear guidance regarding the urgency of referral or when immediate intervention is required. This gap presents challenges in musculoskeletal clinical practice, where physiotherapists must make informed decisions to avoid the misdiagnosis or delayed diagnosis of an underlying serious pathology that may need immediate or urgent medical attention or even surgical intervention [35,36,37,38,39]. Finucane et al. (2020) [8] discuss this issue, noting that RFs are not diagnostic tools but rather predictive guides. They raise a suspicion of underlying pathology but do not provide specific direction on when immediate referral is necessary. For instance, a recent trauma or a history of cancer may raise clinical suspicion, but there is no clear consensus on how these factors should influence the immediate management of the patient. In response to these challenges, the recent literature suggests that certain conditions, such as cardiovascular diseases, may require urgent referral [9]. Feller et al. (2022) [40] highlight signs of acute ischemia, intermittent claudication, rest pain, or ulcers, which necessitate immediate specialist evaluation to prevent severe complications like amputations or heart attacks. These examples illustrate the need for clearer guidance on which RFs necessitate urgent referral, and which may be monitored more conservatively. Correctly identifying conditions requiring urgent referral versus those that can be monitored is crucial for clinical practice. Developing further guidelines to help physiotherapists recognize RFs and determine the urgency of referral could significantly improve patient management and prevent adverse outcomes. Furthermore, variations exist in the level of detail provided for specific RFs and the emphasis on the strength of evidence supporting their use. Some CPGs provide lists of specific RFs categorized by potential pathology [12,17,18], while others focus more on the general principle of identifying warning signs [20,23]. Systematic reviews, including Henschke et al. (2013) [33], Han et al. (2023) [34], and Maselli et al. (2022) [9], highlight the inconsistencies in RF endorsement across different guidelines for the same condition and the variable empirical support for certain RFs. The concept of clustering RFs as a more reliable indicator of serious pathology was emphasized in some guidelines [17,23,28], a notion supported by the findings of Han et al. (2023) [34] regarding vertebral fracture risk, and likely reinforced by the work of Maselli et al. (2022) [9] in the context of thoracolumbar pain. In conclusion, the establishment of a standardized definition of RFs across MSK CPGs is crucial to enhance the clinical reasoning of healthcare professionals with direct patient access [13], ultimately aiming to reduce unnecessary referrals to physicians or hospitals while ensuring the timely and accurate identification of potentially life-threatening and other serious conditions.

### Strengths and Limitations

This comprehensive review adhered to rigorous methodological standards outlined by the JBI, ensuring a systematic and transparent approach to evidence synthesis. The prospective registration of the review protocol on OSF Registries further enhances its transparency and reduces the risk of reporting bias. The well-defined inclusion and exclusion criteria, guided by the PCC framework, ensured the selection of the relevant literature, focusing specifically on the definitions of RFs within MSK care. The comprehensive search strategy, encompassing major databases and the grey literature, aimed to capture a broad spectrum of relevant publications. The dual, independent study selection and data extraction processes, with the resolution of disagreements being mediated by a third experienced reviewer, minimized the potential for selection and extraction bias. Finally, the synthesis of qualitative data involved a systematic comparison of definitions, providing a clear overview of the consistencies and variations present in the literature. Despite these strengths, this study has several limitations. A potential limitation of this review is that five out of the fourteen included studies were published more than 10 years ago, which may affect the contemporaneity of the evidence, given the evolving nature of clinical guidelines and best practices in musculoskeletal care. Nevertheless, this observation may also serve as a call to action for researchers and stakeholders to update their decision-making processes by generating new evidence, rather than continuing to rely on outdated sources, as is currently the case. The focus on CPGs and systematic reviews of CPGs, while targeted to address the research question, may have inadvertently excluded relevant conceptualizations or applications of RFs discussed in primary research articles, expert opinions, or educational materials. Furthermore, the limited number of studies providing explicit and detailed definitions of RFs constrained the depth of our comparative analysis. The inherent heterogeneity in the scope and focus of the included guidelines and reviews, addressing various MSK regions and pathologies, also presented a challenge in achieving a highly granular comparison of specific RF definitions. The restriction to articles in English, Spanish, and Italian may have introduced a language bias, potentially overlooking relevant definitions published in other languages. Finally, the qualitative nature of the data synthesis, while appropriate for the research question, did not allow for the quantitative analysis of the prevalence or diagnostic accuracy associated with specific RF definitions.

## 5. Conclusions

This review underscores a critical gap in MSK physiotherapy practice: the lack of a universally accepted and consistently applied definition of RFs. While CPGs and SRs broadly acknowledge RFs as clinical indicators of potentially serious or life-threatening conditions requiring further medical investigation or referral, the variability in definitions and the absence of clear guidance on referral urgency remain substantial barriers to effective clinical reasoning. The unified definition proposed in this review, based on common elements across the literature, represents a preliminary step toward standardizing RF identification in MSK settings. However, it also highlights current limitations in the diagnostic utility of RFs, particularly regarding their sensitivity, specificity, and the lack of operational guidance for timely decision-making.

To support physiotherapists and other healthcare professionals in direct access settings, future research should focus on the development of evidence-informed, internationally agreed-upon definitions of RFs. These should be integrated into updated CPGs that clearly define when and how RFs warrant urgent referral versus clinical monitoring. Establishing such standards is essential to improve patient safety, reduce unnecessary diagnostic procedures, and ensure the early identification of serious conditions within MSK care.

## Figures and Tables

**Table 1:** Data extraction.

Title	Year	Authors	Journal	Study Design	RF Definition
Clinical guidelines for low back pain: A critical review of consensus and inconsistencies across three major guidelines	2016	O’Connell, N.E. and Cook, C.E. and Wand, B.M. and Ward, S.P. [26]	*Best Practice and Research: Clinical Rheumatology*	Review of clinical practice guidelines	“All three guidelines recommended consideration of potential alternative diagnoses such as specific spinal pathologies…” “…none of the guidelines provide notable detail on the best methods for screening. This reflects a broader inconsistency in the specific detail for red flag screening advocated across guidelines for LBP” “…the Canadian guideline specifies a list of specific indications for MRI including major or progressive neurologic deficit, suspected cauda equina syndrome, progressive severe pain and debility despite non-interventional therapy, severe or incapacitating back or leg pain, and clinical or radiological suspicion of neoplasm or infection”.
Clinical practice guideline for physical therapy assessment and treatment in patients with nonspecific neck pain	2018	Bier, J.D. and Scholten-Peeters, W.G.M. and Staal, J.B. and Pool, J. and van Tulder, M.W. and Beekman, E. and Knoop, J. and Meerhoff, G. and Verhagen, A.P. [27]	*Physical Therapy*	Clinical practice guideline	“Red flags are patterns of sign or symptoms (warning signs) that may indicate serious pathology requiring further medical diagnostics. Red flags may indicate a specific pathology, such as neck pain grade IV” “If RFs are present and not explicable by a known pattern of neck pain, then the patient must be refered” “The evidence supporting the RF for neck is weak and inconsistent because many RF are rather generic (such as enexplained weight loss) and have high false positivity rates”. “RFs are indicators for serious pathological conditions. These conditions include fracture, vertebral artery dissection, spinal cord injury, carvical myelopathy, infection, neoplasm and systemic disease”.
Evaluation of guideline-endorsed red flags to screen for fracture in patients presenting with low back pain	2019	Parreira, P.C.S. and Maher, C.G. and Traeger, A.C. and Hancock, M.J. and Downie, A. and Koes, B.W. and Ferreira, M.L. [28]	*British Journal of Sports Medicine*	Systematic review of guidelines	The authors describe the use of red flags to identify fractures in LBP citing several examples in the text “Red flags are clinical indicators—signs, symptoms, or patient history factors—that raise suspicion of a serious underlying pathology in patients presenting with low back pain, such as vertebral fracture, malignancy, infection, or inflammatory disease. Their primary purpose is to assist clinicians in identifying individuals who may require further diagnostic evaluation or referral for medical assessment. Commonly endorsed red flags for vertebral fracture include older age, history of significant trauma, prolonged corticosteroid use, and osteoporosis. However, many red flags—such as night pain or female gender—lack robust diagnostic evidence and may lead to unnecessary imaging or false positives when used in isolation. The authors emphasize that combinations of red flags are more diagnostically useful than individual indicators, as the presence of multiple red flags significantly increases the likelihood of serious pathology”.
Evidence based practice guidelines for management of low back pain: Physical Therapy implications	2011	Ladeira, C.E. [17]	*Revista Brasileira de Fisioterapia*	Systematic Review of guidelines	Red flags were designed to identify patients with LBP associated with specific spine pathologies that require physician specialist referral. Any patient who presented with red flags indicating suspicion of cancer, infection, cauda equina syndrome, spondyloarthritis, spinal fracture, visceral (gastrointestinal and genitourinary) referred pain, and abdominal aortic aneurism need to be sent to a specialist. Red flags for patients with low back pain: cauda equina syndrome: saddle anesthesia or paresthesia, perianal/perineal sensory loss; positive straight leg raise testing, multiple motor deficits; bowel/bladder dysfunction, fecal/urinary incontinence; severe (paralysis rather than paresis) or bilateral neurological compromise. Spinal fracture recent violent trauma (fall from great height, car accident); minor trauma in patients with a history of osteoporosis, older age; structural bone deformity, prolonged corticosteroid use; severe central back pain relieved by lying down. Cancer or infection age above 50 and below 20 years old; constitutional symptoms (e.g., fever, weight loss, chills, malaise); history of cancer (malignancies), thoracic spine pain; recent bacterial infection (e.g., urinary tract, respiratory tract); immune depression (e.g., HIV *, chemotherapy), intravenous drug abuse; prolonged use of corticosteroids, recent puncture wound or surgery, diabetes, spinal tenderness to percussion; recent or fast developing spine deformity (e.g., scoliosis); non-mechanical (e.g., not better when lying down) or progressive pain, failure to improve with treatment in 4 to 6 weeks, unremitting night time pain. Abdominal aortic aneurysm age over 60, history of cardiovascular disease (e.g., myocardial infarct or stroke); pulsating mass on the abdomen, leg pain, thoracic pain; absence of aggravating features; spondyloarthritis age less than 45 years old, morning stiffness improved with exercise; alternating buttock pain, significant and persistent lumbar flexion restriction (positive Schober’s test); awakening because of back pain during second part of night; oligoarthritis or polyarthritis, skin rashes, diarrhea, hypersensitivity to NSAIDs *. Gastrointestinal or genitourinary abdominal or flank pain/tenderness, rebound tenderness, costo-vertebral angle tenderness; reduced urine stream, reduced stool caliper, burning during urination, abnormal urine or stool coloration/smell; diarrhea, constipation, anuria, oliguria, polyuria; abnormal menses, dyspareunia, painful erection; patients presenting with cauda equina syndrome and abdominal aortic aneurism required immediate referral and possibly emergency care. Patients with high fever (>38 °C or 100.4 °F) lasting longer than 48 h, progressive neurological signs and symptoms (i.e., paresis to paralysis, peripheralization of pain), or unrelenting night pain not relieved by postural changes required urgent consultation within 24 h. A single red flag (e.g., age over 50) was not enough to indicate specialist referral, but a patient presenting with a cluster of red flags (e.g., age over 50, non-mechanical pain, thoracic spine pain) should definitely be referred for medical consultation.
Management of neck pain and associated disorders: A clinical practice guideline from the Ontario Protocol for Traffic Injury Management (OPTIMa) Collaboration	2016	Cote, P. et al. [18]	*European Spine Journal*	Guideline	“Recommendation 1: Clinicians should rule out major structural or other pathologies as the cause of NAD. Evaluation Clinicians should conduct a clinical evaluation to rule out major structural or other pathologies (NAD grade IV) as the cause of signs and symptoms. The Canadian C-spine Rule should be used to rule out cervical spine fractures and dislocations associated with acute trauma. The presence of risk factors for serious pathologies (also termed ‘red flags’) identified during the history/examination warrants further investigation and referral to the appropriate healthcare professional. Clinicians should assess for neurological signs (decreased deep tendon reflexes, muscle weakness, sensory deficits). NAD III refers to neck pain associated with clear clinical evidence of neurologic signs (decreased deep tendon reflexes, weakness, or sensory deficits) on physical examination. Once major pathology has been ruled out, clinicians should classify the grade of NAD as grade I, II, or III; as recent or persistent; and the patient should receive the appropriate evidence-based interventions. Figures 1 and 2 (rule out risk factors for serious pathologies—red flags) and Table 4 (risk factors for serious pathology—red flags) for neck pain): Cancer (history of cancer, unexplained weight loss, nocturnal pain, age > 50 years), vertebral infection (fever, intravenous drug use, recent infection), osteoporotic fractures (history of osteoporosis, use of corticosteroid, older age), traumatic fractures (positive Canadian C-Spine Rule), myelopaty-severe/progressive neurological deficits (painful stiff neck, arm pain and weakness sensory changes in lower extremities, motor weakness and atrophy, hyper-reflexia, spastic gait), carotid/vertebral artery dissection (sudden and intense onset of headache or neck pain), brain hemorrage/mass lesion (sudden and intense onset of headache), inflammatory arthritis (morning stiffness, swelling in multiple joints)”.
Most red flags for malignancy in low back pain guidelines lack empirical support: A systematic review	2017	Verhagen, A.P. and Downie, A. and Maher, C.G. and Koes, B.W. [25]	*Pain*	Systematic Review of guidelines	We defined red flags as signs or symptoms collected in the clinical assessment signaling underlying serious pathology that requires attention (Merriam-Webster dictionary); Table 1 (number of guidelines endorsing red flag for malignancy for the management of low back pain in primary care): history of malignancy/cancer; unexplained/unintentional) weight loss; atypical pain either increasing at night or at rest or pain at night that is not eased by a prone position (or increasing in supine position); older age; either just older age or more specifically over 50 years; malaise; failure to improve with treatment (>4–6 weeks)/seeking medical care last month; strong clinical suspicion; fever; reduced appetite; rapid fatigue; progressive symptoms; multiple cancer risk factors; paraparesis. Red flags not endorsed in guidelines: duration of the complaint > 1 month; disturbed balance, weakness of limbs.
New guidelines for the management of migraine in primary care	2002	Dowson, A.J. and Lipscombe, S. and Sender, J. and Rees, T. and Watson, D. [19]	*Current Medical Research and Opinion*	Guideline	“Sinister headache: Primary care physicians need a means of identifying patients with rare or secondary (sinister) headaches who are best referred to a specialist. Has the pattern of your headache changed over the last 6 months? (This is designed to alert the physician to sinister headache conditions. A new or different headache mandates a thorough diagnostic approach, while a stable headache pattern provides reassurance to the physician and patient). Table 1: The exclusion of secondary headaches, by a search for ‘headache alarms’, by history taking or physical examination. Table 2: Sinister headache should be excluded. Sinister headaches tend to appear de novo in young children or mature adults, or present as a change in character compared with older patients’ usual headache attacks. They are new-onset, acute headaches that are associated with a range of other symptoms (e.g., rash, neurological deficit, vomiting and pain or tenderness). Signs of neurological change or deficit do not disappear when the patient is pain-free between headache attacks. They may also be associated with an accident or head injury, infection or hypertension. A full neurological examination is essential if sinister headache is suspected”.
Nonarthritic hip joint pain: Clinical practice guidelines linked to the international classification of functioning disability and health from the orthopaedic section of the american physical therapy association	2014	Enseki, K. and Harris-Hayes, M. and White, D.M. and Cibulka, M.T. and Woehrle, J. and Fagerson, T.L. and Clohisy, J.C. and Godges, J. [20]	*Journal of Orthopaedic and Sports Physical Therapy*	Clinical Practice Guideline	In the context of clinical practice guidelines for nonarthritic hip joint conditions, the information provided suggests that the authors define a red flag as follows: “Red flag is a clinical indicator suggesting the potential presence of a condition more serious or unrelated to nonarthritic hip joint pain. It should be suspected when the patient’s history, reported activity limitations, or impairments in body function and structure are inconsistent with the typical presentation of nonarthritic hip disorders, or when symptoms do not improve with interventions aimed at normalizing identified impairments. In such cases, clinicians should broaden their diagnostic approach to consider alternative or serious pathologies such as infection, neoplasm, gynecological disorders, stress fractures, or systemic diseases, which require different diagnostic and therapeutic strategies”.
Physiotherapy in hip and knee osteoarthritis: Development of a practice guideline concerning initial assessment treatment and evaluation	2011	Peter, W.F.H. et al. [21]	*Acta Reumatologica Portuguesa*	Clinical Practice Guideline	In this context, the authors state that a “red flag” “is a clinical sign or symptom that indicates a potentially serious underlying condition requiring immediate attention or referral to a specialist. These signs or symptoms suggest the need for further investigation beyond typical musculoskeletal issues, such as infections, malignancies, or severe joint conditions. Specifically, in the case of hip and knee osteoarthritis patients, red flags include: A warm, swollen (red) knee joint (potential bacterial infection) Swelling in the groin (possible malignancy) Severe blocking of the knee joint (indicating significant joint dysfunction) Physiotherapists are responsible for identifying these red flags during their assessment and referring patients for further medical evaluation as needed”.
Physiotherapy in rheumatoid arthritis: development of a practice guideline	2011	Hurkmans EJ et al. [22]	*Acta Reumatologica Portuguesa*	Clinical Practice Guideline	In the context of rheumatoid arthritis, the authors defined “red flags” as specific signs and symptoms that indicate a serious underlying condition or complication, potentially requiring urgent medical attention or referral to a specialist. These red flags may signal acute or worsening health issues that go beyond typical RA symptoms, suggesting the need for further evaluation.
Red flags presented in current low back pain guidelines: a review	2016	Verhagen, A.P. and Downie, A. and Popal, N. and Maher, C. and Koes, B.W. [24]	*European Spine Journal*	Systematic Review of clinical practice guidelines	“To identify and compare the red flag recommendations in current guidelines for the detection of medically serious pathology in patients presenting with low back pain. The authors included 16 discrete guidelines for the management of patients with low back pain in the primary care setting presenting 46 different red flags for the four main categories of serious underlying pathologies (malignancy, fracture, infection and cauda equina syndrome) [1]. Malignancy = History of malignancies/cancer, (Unexplained/unintentional) Weight loss, (Increasing) Pain at night, (Continuous) Pain at rest, Pain at multiple sites, Pain over 1 month (duration), Pain at night that is not eased by a prone position (or increasing in supine position), Failure to improve with treatment (4–6 weeks), Age over 50 years/Old age, Elevated erythrocyte sedimentation (ESR), General malaise, Multiple cancer risk factors, Strong clinical suspicion, Reduced appetite, Rapid fatigue, Progressive symptoms, Fever, Paraparesis, Age over 50 (over 65), first episode of severe back pain and history of cancer/carcinoma in the last 15 years, unexplained weight loss, failure of conservative care (4 weeks) [2]. Fracture = (History of) Major/significant trauma, (Systemic) Use of steroids, Osteoporosis, Female gender, Age over 50/Age over 60/Older age (over 70), Sudden onset (of pain), Loading pain, Minor trauma, Fracture in history/previous fractures, Low body weight, Increased thoracic kyphosis, Structural deformity, Minor trauma (if age over 50, history of osteoporosis and taking corticosteroids), Severe onset of pain (with minor trauma, age over 50, prolonged steroid intake or structural deformity) [3]. Infection = Fever ?38 °C, Use of corticosteroids or immunosuppressant therapy, Intravenous drug abuse/drug addiction, Immunodeficiency/AIDS, Urinary tract infection, Pain with recrudescence at night, Intense night pain (and rest pain), Bone tenderness over the lumbar spinous process, Previous back surgery, Previous bacterial infections, Penetrating wound, Reduced appetite, Rapid fatigue, Impaired immune system, Underlying disease process [4]. Cauda Equina Syndrome (CES) = Saddle anesthesia/perineal numbness, (Sudden onset) Bladder dysfunction (e.g., urinary retention, overflow incontinence), Sphincter disturbance/reduced tonus, Progressive weakness in lower limbs/lower motor neuron weakness (Wide) Spread sensory deficit (in lower limbs), Gait disturbance/abnormality, Fecal incontinence, Pain (radiating) in both legs, Sciatica Red flags unrelated to specific diseases: Pain: Onset of pain <20 or >50 years old, Constant progressive non-mechanical pain, No pain relief with bed rest, Thoracic or abdominal pain, (Continuous) Pain at rest, (Increasing) Pain at night, Pain increase in flexion, Increasing pain despite treatment, Pain at night that is not eased by a prone position (or increasing in supine position); Malignancy: History of malignancies/cancer, (Unexplained/unintentional) Weight loss, General malaise, Elevated erythrocyte sedimentation (ESR), Age over 50 years; Fracture: (History of) Major/significant trauma, (Structural spinal) deformity, (Systemic) Use of steroids, Osteoporosis; Infection: Fever ?38 °C, Intravenous drug abuse/drug addiction, Use of corticosteroids or immunosuppressant therapy, Immunodeficiency/HIV/AIDS; Cauda Equina Syndrome (CES): Saddle anesthesia/perineal numbness, (Sudden onset) Bladder dysfunction (e.g., urinary retention, overflow incontinence), (Wide) Spread sensory deficit (in lower limbs), Progressive weakness in lower limbs/lower motor neuron weakness, Gait disturbance/abnormality; Other: Significant limitation of lumbar flexion, Not flexion of 5th lumbar spine, Morning stiffness”.
Spinal Manipulative Therapy and Other Conservative Treatments for Low Back Pain: A Guideline From the Canadian Chiropractic Guideline Initiative	2018	Bussières, A. E., Stewart, G., Al-Zoubi, F., Decina, P., Descarreaux, M., Haskett, D., Hincapié, C., Pagé, I., Passmore, S., Srbely, J., Stupar, M., Weisberg, J., & Ornelas, J. [29]	*Journal of Manipulative and Physiological Therapeutics*	Guideline	Signs of serious structural or systemic pathologies: history of malignancy and strong clinical suspicion; older age; Prolonged corticosteroid use (increased risk of vertebral fractures); major or significant trauma (high-impact events); presence of contusion or abrasion.
The Belgian national guideline on low back pain and radicular pain: Key roles for rehabilitation, assessment of rehabilitation potential and the PRM specialist	2020	Van Wambeke, P. and Desomer, A. and Jonckheer, P. and Depreitere, B. [23]	*European Journal of Physical and Rehabilitation Medicine*	Clinical practice guideline	It is of outmost importance to perform a thorough evaluation of the complaints of the patient each time and exclude signs and/or symptoms of possible serious underlying pathology (identified as red flags) and to focus hereby on clusters of red flags. The actual guideline does not address the further management of these pathologies.
Red flags for potential serious pathologies in people with neck pain: a systematic review of clinical practice guidelines	2024	Feller, D. et al. [12]	*Archives of Physiotherapy*	Systematic Review of clinical practice guidelines	“Authors identified 29 guidelines in which they found 114 red flags: Fracture (Canadian C-Spine Rule [a clinical decision rule used to determine the need for radiography in patients with neck trauma], history of trauma, history of osteoporosis, use of corticosteroids, older age >50/60); Cancer (history of cancer, unexplained weight loss, age >50/60, failure to improve after one month of conservative care, pain that worsens at night, unrelenting pain, not relieved by rest); Spinal Infection (fever, history of recent infection, immunosuppression, drug use, HIV positivity, night sweats or chills); Myelopathy (spasticity, gait disturbances, clumsiness of hands, hyperreflexia, bowel or bladder dysfunction); Spinal Cord Injury (neurological deficits following trauma); Cervical Artery Dissection (recent neck trauma or sudden neck movement, severe unilateral headache or neck pain, signs of stroke or transient ischemic attack, visual disturbances, dizziness or vertigo, horner’s syndrome); Intracranial Pathology (severe, sudden-onset headache, loss of consciousness, neurological signs like visual loss, diplopia, or cranial nerve dysfunction); Inflammatory Arthritis (swelling in multiple joints, morning stiffness lasting more than 30 min); Other Systemic Diseases (fatigue, general malaise, unexplained fever, skin changes, history of autoimmune disease); Non-specific (General) Red Flag (fever, night pain, pain not mechanical in nature, rapidly progressive symptoms, failure to improve with treatment)”.

## Data Availability

Data are available upon reasonable request to the corresponding author.

## Appendix A. String Research Strategies

### Appendix A.1. PubMed

(“Musculoskeletal Diseases”[Mesh]) OR (“Musculoskeletal Diseases”) OR (“musculoskeletal condition*”) OR (“orthopedic disease*”) OR (“orthopedic condition*”) OR (“Cumulative Trauma Disorders”[Mesh]) OR (“Cumulative Trauma Disorders”) OR (“musculoskeletal injury”) OR (“orthopedic injury”) OR (“Musculoskeletal Pain” [Mesh]) OR (“Musculoskeletal Pain”) OR (“orthopedic pain”) OR (“mechanical pain”) OR (“comorbidity”) OR (“pain referred complications”) OR (“musculoskeletal complications”)) OR (“pathological conditions, signs and symptoms” [Mesh]) OR (“pathological conditions, signs and symptoms”) OR (“malingering” [Mesh]) OR (“malingering”) OR (“acute pain” [Mesh]) OR (“Pain” [Mesh]) OR (“Pain, Referred” [Mesh]) OR (“Pain, Referred”) OR (“life-threatening patholog*”) OR (“internal medicine disease*”) OR (“visceral injury”) OR (“Visceral Pain” [Mesh]) OR (“visceral condition*”) OR (“Visceral Pain”) AND (“Diagnosis, Differential” [Mesh]) OR (“Diagnosis differential”) OR (“Clinical Decision-Making” [Mesh]) OR (“Clinical Decision Making”) OR (“Clinical Decision Rules” [Mesh]) OR (“Clinical Decision Rules”) OR (“clinical practice decision”) OR (“red flag screening”) OR (“red flag”) AND (“diagnosis”) OR (“physical examination”) OR (“Physical Examination” [Mesh]) OR (“referral and consultation”) OR (“diagnostic techniques and procedures” [Mesh]) OR (“prodromal symptoms” [Mesh]) OR (“referral and consultation” [Mesh]) OR (“specialist referral”) OR (“secondary care center *” [Mesh]) OR (“physician practice pattern”) OR (“Practice Patterns, Physicians’“ [Mesh]) OR (“patient care team”) OR (“patient care team” [Mesh]) OR (“pain etiology”) OR (“Pain, diagnosis”) OR (“pain, physiopathology”) OR (“Diagnostic Tests, Routine” [Mesh]) OR (“medical history taking”) OR (“medical history taking” [Mesh]) OR (”musculoskeletal diagnosis”) OR (“diagnosis” [Subheading]) OR (“pain referred diagnosis”) OR (“pain referred physiopathology”) OR (“critical pathways” [Mesh]) OR (“visceral pain, etiology”) AND (“Physical Therapy Modalities” [Mesh]) OR (“Physical Therapists” [Mesh]) OR (“Physical Therapists”) OR (“physical therapy specialty” [Mesh]) OR (“Physiotherapy”) OR (“Physiotherapists”) AND (“clinical practice guidelines”) OR (“guideline*”)

### Appendix A.2. Web of Science

((((ALL = ((“Musculoskeletal Diseases”) OR (“Musculoskeletal Diseases”) OR (“musculoskeletal condition*”) OR (“orthopedic disease*”) OR (“orthopedic condition*”) OR (“Cumulative Trauma Disorders”) OR (“Cumulative Trauma Disorders”) OR (“musculoskeletal injury”) OR (“orthopedic injury”) OR (“Musculoskeletal Pain”) OR (“Musculoskeletal Pain”) OR (“orthopedic pain”) OR (“mechanical pain”) OR (“comorbidity”) OR (“pain referred complications”) OR (“musculoskeletal complications”) OR (“pathological conditions, signs and symptoms”) OR (“pathological conditions, signs and symptoms”) OR (“malingering”) OR (“malingering”) OR (“acute pain”) OR (“Pain”) OR (“Pain, Referred”) OR (“Pain, Referred”) OR (“life-threatening pathology *”) OR (“internal medicine disease*”) OR (“visceral injury”) OR (“Visceral Pain”) OR (“visceral condition*”) OR (“Visceral Pain”))) AND ALL = ((“Diagnosis, Differential”) OR (“Diagnosis differential”) OR (“Clinical Decision-Making”) OR (“Clinical Decision Making”) OR (“Clinical Decision Rules”) OR (“Clinical Decision Rules”) OR (“clinical practice decision”) OR (“red flag screening”) OR (“red flag”))) AND ALL = ((“diagnosis”) OR (“physical examination”) OR (“Physical Examination”) OR (“referral and consultation”) OR (“diagnostic techniques and procedures”) OR (“prodromal symptoms”) OR (“referral and consultation”) OR (“specialist referral”) OR (“secondary care center*”) OR (“physician practice pattern”) OR (“Practice Patterns, Physicians’“) OR (“patient care team”) OR (“patient care team”) OR (“pain etiology”) OR (“Pain, diagnosis”) OR (“pain, physiopathology”) OR (“Diagnostic Tests, Routine”) OR (“medical history taking”) OR (“medical history taking”) OR (”musculoskeletal diagnosis”) OR (“diagnosis” [Subheading]) OR (“pain referred diagnosis”) OR (“pain referred physiopathology”) OR (“critical pathways”) OR (“visceral pain, etiology”))) AND ALL = ((“Physical Therapy Modalities”) OR (“Physical Therapists”) OR (“Physical Therapists”) OR (“physical therapy specialty”) OR (“Physiotherapy”) OR (“Physiotherapists”))) AND ALL = ((“clinical practice guidelines”) OR (“guideline *”))
